# Sarcoidosis-associated pulmonary hypertension: Clinical features and outcomes in Arab patients

**DOI:** 10.4103/1817-1737.62471

**Published:** 2010

**Authors:** Esam H. Alhamad, Majdy M. Idrees, Mohammed O. Alanezi, Ahmad A. AlBoukai, Shaffi Ahmad Shaik

**Affiliations:** *Department of Medicine, Pulmonary Division, King Khalid University Hospital, King Saud University, Riyadh, Saudi Arabia*; 1 *Department of Medicine, Pulmonary Medicine, Riyadh Military Hospital, Riyadh, Saudi Arabia.*; 2 *Department of Medicine, Pulmonary Medicine, King Abdulaziz Medical City, Riyadh, Saudi Arabia*; 3 *Department of Radiology and Medical Imaging, King Khalid University Hospital, King Saud University, Riyadh, Saudi Arabia.*; 4 *Department of Family and Community Medicine, King Khalid University Hospital, King Saud University, Riyadh, Saudi Arabia.*

**Keywords:** Computed tomography, echocardiography, pulmonary function test, pulmonary hypertension, sarcoidosis

## Abstract

**BACKGROUND:**

Pulmonary hypertension (PH) occurs in many patients with interstitial lung disease, including sarcoidosis. We explored the frequency, clinical characteristics and outcomes of PH in Arab patients diagnosed with pulmonary sarcoidosis.

**METHODS:**

A retrospective study in three tertiary hospitals was performed on 96 patients who underwent Doppler echocardiography. Demographic and clinical characteristics, physiological studies and computed tomography (CT) results were collected, and compared between patients with and without PH.

**RESULTS:**

Twenty (20.8%) patients were found to have PH. Patients with PH were more likely to be symptomatic (cough, *P* = 0.008; dyspnea, *P* = 0.04), to have an advanced radiographic stage (*P* = 0.001), and to be receiving systemic therapy (*P* = 0.001), compared to those without PH. Physiological data including pulmonary function test parameters, arterial blood gas levels and oxygen saturation at rest and after exercise were all significantly lower in patients with PH compared to those without PH. Comparison of CT patterns between patients with and without PH showed significant differences in the frequencies of ground-glass opacity (61.5 vs. 28.8%, *P* = 0.032) and fibrosis (76.9 vs. 44.2%, *P* = 0.035). In total, four patients died during the study period, including three with evidence of PH.

**CONCLUSIONS:**

The frequency of PH in the present study was 20.8%. Clinical, physiologic and radiographic characteristics appeared to differentiate patients with PH from those without PH. The presence of PH contributed to poor outcomes in patients with pulmonary sarcoidosis.

Sarcoidosis is a granulomatous disease characterized by manifestation in diverse organs and significant heterogeneity in frequency, clinical characteristics and severity across different ethnic groups and genders. Although the original disease description appeared over 130 years ago, the etiology of sarcoidosis remains elusive. Most patients either show spontaneous remission or have stable disease, but up to one-third of patients develop chronic progressive disease.[1] Pulmonary hypertension (PH) is a well-recognized complication of various chronic respiratory diseases, and the presence of PH has a significant impact on survival among patients with chronic obstructive lung disease,[2] scleroderma[3] and idiopathic pulmonary fibrosis.[4] Furthermore, the presence of PH is considered one of the most important (negative) prognostic factors in patients with sarcoidosis.[5,6] Very few studies have focused on sarcoidosis in Arab patients[7–10] and there have been no reports to date on the frequency of PH within the Arab population.

This retrospective study was conducted to explore clinical characteristics and outcomes in Arab patients diagnosed with pulmonary sarcoidosis and PH.

## Methods

### Study population

We retrospectively reviewed the medical records of all sarcoidosis patients seen in the outpatient pulmonary clinics of three study centers (King Khalid University Hospital, King Abdulaziz Medical City and Riyadh Military Hospital) in Riyadh, Saudi Arabia, between January 2001 and December 2008. The investigation was approved by the Ethics Committee of each participating hospital. Sarcoidosis was diagnosed based on the latest American Thoracic Society (ATS), European Respiratory Society (ERS) and World Association of Sarcoidosis and Other Granulomatous Disorders (WASOG) criteria.[11] Although these guidelines appeared only in 1999, we verified that all patients included in the present study fulfilled all designated criteria. Patients displaying evidence of mycobacterial or fungal infection and those with histories of ingestion of drugs or agents causing granulomatous lung disease were excluded. Extrapulmonary organ involvement was defined according to the criteria established by Judson and colleagues.[12] One hundred and sixteen sarcoid patients were candidates for inclusion in the study. To be included, each patient had to have 1) available two-dimensional echocardiographic data, 2) pulmonary sarcoidosis diagnosed by histopathology, 3) age >20 years and 4) Arab ethnic origin. Ninety six patients met these criteria. Data collected included patient demographics, symptoms, details of comorbid illnesses, sarcoidosis stage using the modified Scadding[13] classification system to stage chest radiography (CXR) findings, measurement of room air arterial blood gas levels, treatment, pulmonary function test results and 6-min walk test (6MWT) data. All tests were performed close to the date on which echocardiography data were recorded. Pulmonary function tests included spirometry and lung volume measurement by body plethysmography, performed according to the recommendations of the American Thoracic Society.[14] The 6MWT was conducted in accordance with ATS guidelines.[15] All patients exhibited resting oxygen saturation (SpO_2_) >88% at the beginning of the walk test. Heart rate, blood pressure, oxygen saturation and Borg dyspnea index[16] were recorded at the beginning and end of the 6-min walk. Available 6MWTs (*n* = 48) were performed according to ATS guidelines.[15] Computed tomography (CT) findings were assessed for the presence of the following recognized CT patterns:[17] (1) mediastinal and/or hilar lymph node enlargement; (2) ground-glass opacity; (3) consolidation; (4) nodules <3 cm in diameter; (5) thickening of bronchovascular bundles; (6) linear opacity, including interlobular septal lines and interstitial thickening and (7) features indicating scarring and fibrosis (grouped together) that included traction bronchiectasis, honeycombing, cysts and/or volume loss. Diagnosis of PH was based on Doppler echocardiography and defined as an estimated right ventricular systolic pressure (RVSP) of th ≥40 mm Hg in the absence of left ventricular dysfunction (i.e., an ejection fraction under 50%), ischemic heart disease (manifested by regional wall motion abnormalities) or valvular heart disease. Right ventricular systolic pressure was estimated based on the modified Bernoulli equation:[18] RVSP = transtricuspid gradient + right atrial pressure (RAP), where transtricuspid gradient is 4*v*^2^ (*v* = peak velocity of tricuspid regurgitation in meter per second) and RAP was estimated to be 5, 10 and 15 mm Hg based on the respiratory variation of the inferior vena cava.[19]

### Statistical analysis

Data were entered into MS Excel and analysed using the Statistical Software Package for Social Sciences (SPSS pc+ version 13.0; SPSS, Inc; Chicago, IL). Descriptive statistics (i.e., means, standard deviations, and percentages) were used to summarize both continuous and categorical variables. The relationship between any two continuous variables was explored using Pearson's correlation coefficient. Student's *t*-test for independent samples was applied to compare mean values of continuous variables. The chi-squared test was used to compare proportions of categorical study variables with those of categorical outcome variables. In all analyses, two-tailed *P* values <0.05 were considered significant.

## Results

The study sample comprised 96 patients, and the estimated right ventricular systolic pressure was ≥40 mm Hg in 20 (20.8%) patients. Demographic and clinical data are summarized in Table 1.

**Table 1:** Demographic and clinical characteristics*

Variables	PH present *n* = 20	PH absent *n* = 76	*P* value
Age, years	49.2 ± 14.2	50.8 ± 13.7	NS
Male/Female, no.	3/17	29/47	NS
Disease duration, months	96.9 ± 57.5	117.1 ± 75.2	NS
Smoker/Nonsmoker, no.	4/16	10/66	NS
Symptoms	
Cough, no.	19	51	0.008
Dyspnea, no.	19	57	0.04
Chest radiographic stage,	-/2/6/3/9	5/22/34/9/6	0.001
no. (0/I/II/III/IV)	
Receiving corticosteroids and/	20	49	0.001
or immunosuppressive therapy	

* Data are presented as means ± SDs, unless otherwise indicated. NS = not significant

Comparisons between patients with and without PH showed no differences in mean age (*P* = 0.64) or body mass index (BMI) (28.3 + 6.2 vs. 28.8 + 5.8 kg/m^2^, *P* = 0.75). A higher female-to-male ratio was noted among patients with PH, compared to those without PH (5.7: 1 vs. 1.5: 1).

Comorbid illnesses were similar, with no marked differences observed between groups (diabetes mellitus, *P* = 0.84; hypertension, *P* = 0.55 and ischemic heart disease, *p* = 0.53). Patients with PH were more likely to show an advanced radiographic stage (*P* = 0.001), and to be receiving corticosteroids alone or in combination with immunosuppressive therapy, compared to those without PH (*P* = 0.001).

Extrapulmonary organ involvement did not differ between patients with PH and those without PH [eye (*n* = 9), liver (*n* = 8), skin (*n* = 4), spleen (*n* = 2), kidney (*n* = 2), bone marrow (*n* = 2), central nervous system (*n* = 1), bone (*n* = 1), heart (*n* = 1) and superficial lymph nodes (*n* = 1)]. In addition, there was no significant between-group difference in mean calcium level (PH present: 2.31 + 0.15 mmol/l; PH absent: 2.32 + 0.16 mmol/l, *P* = 0.91).

**Figure 1:**
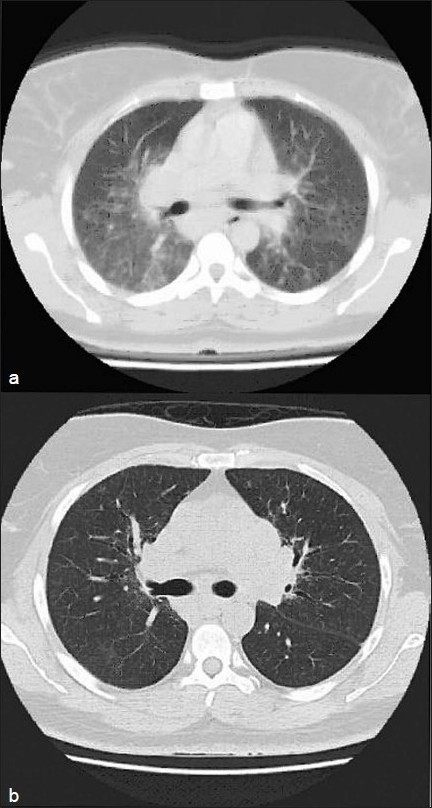
Patient 2: (a) HRCT scan showing widespread nodules with patchy areas of ground glass attenuation. (b) HRCT scan obtained at the 5-year follow-up showing regression of ground glass attenuation with treatment. Clinical characteristics of the patient are shown in Table 4

**Figure 2:**
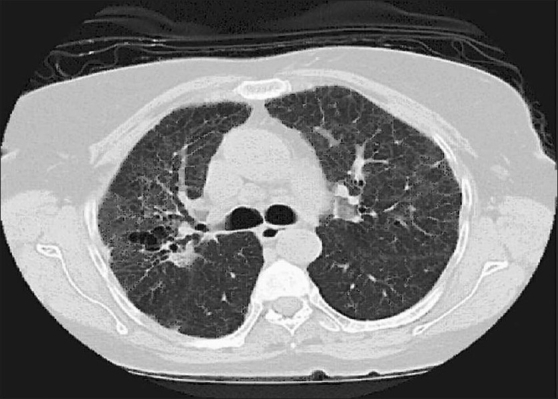
Patient 3. HRCT scan obtained at the level of tracheal bifurcation demonstrating fibronodular pattern with traction bronchiectasis. Mediastinal lymph nodes enlargement are seen. Clinical characteristics of the patient are shown in Table 4

**Figure 3:**
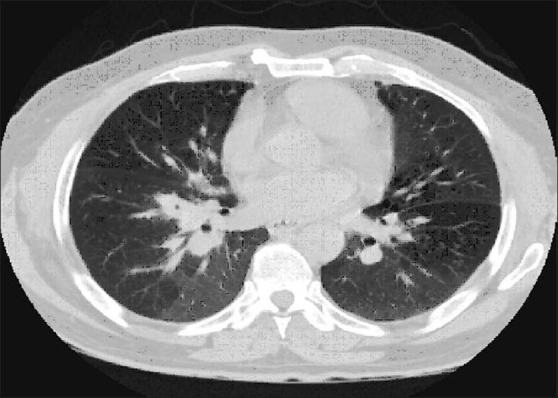
Patient 4. HRCT scan at the mid lung zones showing conglomeration pattern with background diffuse hyperinflation. Clinical characteristics of the patient are shown in Table 4

**Figure 4:**
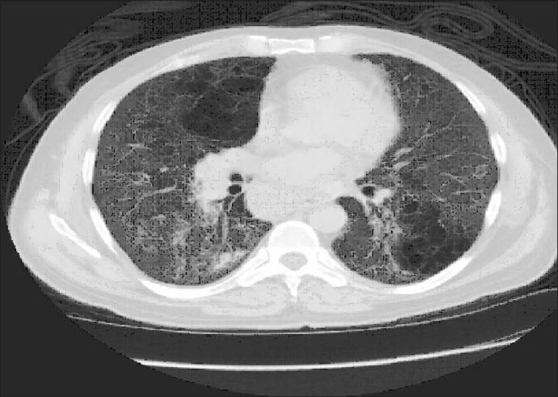
Patient 5. HRCT scan at the level of the left atrium demonstrating diffuse ground glass attenuation with fine reticulation and cystic changes. Lymph node enlargement involving the azygoesophageal recess is seen. Clinical characteristics of the patient are shown in Table 4

Physiologic parameters including absolute values and predicted percentages of FVC, FEV_1_ and TLC were significantly lower in patients with PH, compared to those without PH [Table 2]. Furthermore, patients with PH were more likely to be hypoxic and hypercapnic compared to those without PH. Six-minute walk distance values were available for 13 patients with PH and 35 patients without PH. The mean distance walked was shorter for patients with PH compared to those without PH, although the difference did not reach statistical significance (*P* = 0.07). Furthermore, significant differences in oxygen saturation at rest and after exercise were noted between the groups. Notably, nine patients (five of stage II, two of stage III and two of stage IV) without PH displayed desaturation to <90% during the 6MWT. CT data were available for 13 patients with PH and 52 without PH [Table 3]. When CT variables were compared between patients with and without PH, statistically significant differences were noted in ground-glass opacity (61.5 vs. 28.8%, *P* = 0.032) and fibrosis (76.9 vs. 44.2%, *P* = 0.035); other CT parameters showed no marked differences. Data on right heart catheterization (RHC) were available for five patients who received echocardiography on the same day, and pulmonary function tests, 6MWT and high-resolution CT (HRCT) scan within 7 days of RHC [Table 4] [Figures 1–4]. In comparison to the results of RHC, two patients were found to have false-negative Doppler echocardiography studies; however, they were not included in the PH group.

**Table 2:** A comparison of physiological data between patients with and without PH*

Variables	PH present *n* = 20	PH absent *n* = 76	*P* value
FVC, L	1.6 ± 0.7	2.6 ± 0.9	<0.0001
FVC, % predicted	56.9 ± 21.3	79.5 ± 20.9	<0.0001
FEV_1_, L	1.4 ± 0.6	2.1 ± 0.8	<0.0001
FEV_1_% predicted	57.9 ± 21.5	78.2 ± 21.0	<0.0001
TLC, L	3.2 ± 1.2	4.0 ± 1.1	0.03
TLC, % predicted	67.1 ± 17.9	79.6 ± 19.2	0.031
PaO_2_, mmHg	62.6 ± 13.4	73.4 ± 11.9	0.002
PaCO_2_, mmHg	42.4 ± 8.7	36.8 ± 5.3	0.002
6MWD, m†	313.2 ± 63.1	356.4 ± 71.6	0.071
Initial SpO_2_, %	93.0 ± 3.0	95.7 ± 2.21	0.001
Lowest SpO_2_, %	84.6 ± 10.6	92.1 ± 5.8	0.003

* Data are presented as means ± SDs, unless otherwise indicated. FVC = forced vital capacity, FEV_1_ = forced expiratory volume in 1 s, TLC = total lung capacity, PaO_2_ = partial pressure of oxygen, PaCO_2_ = partial pressure of carbon dioxide, , SpO_2_ = oxygen saturation by pulse oximetry,

† 6MWD = 6-minute walk distance (PH present *n* = 13, PH absent *n* = 35)

**Table 3:** Comparison of CT findings*

Variables		PH present *n* = 13	PH absent *n* = 52	*P* value
Lymph node enlargement	(yes/no)	6/7	27/25	NS
Nodules	(yes/no)	7/6	26/26	NS
Consolidation	(yes/no)	1/12	2/50	NS
Linear opacity	(yes/no)	4/9	15/37	NS
Ground-glass opacity	(yes/no)	8/5	15/37	0.032
Bronchovascular bundles thickening	(yes/no)	4/9	12/40	NS
Scarring and fibrosis	(yes/no)	10/3	23/29	0.035

* NS = not significant

**Table 4:** Clinical characteristics of five patients with available right heart catheterization

Variables	Patient 1	Patient 2	Patient 3	Patient 4	Patient 5
Age, years	37	40	56	60	59
Gender	Female	Female	Female	Male	Male
Disease duration, years	5	4	8	8	5
Smoking history	Never smoker	Former smoker	Never smoker	Never smoker	Never smoker
Extra-pulmonary organ	Eye	None	None	None	None
Involvement					
Treatment	Azathioprine	Corticosteroids	Corticosteroids	Corticosteroids	Corticosteroids
RSVP, mmHg (Echocadiography)	34	48	28	58	83
sPAP, mmHg	41	47	40	52	55
mPAP, mmHg	31	37	25	30	38
PCWP, mmHg	15	11	6	14	12
PVR Wood units	3.0	5.4	2.9	3.07	5.6
CO, I/min	5.29	4.8	6.47	5.2	4.64
FVC, %predicted	66.8	77.6	86.7	56.5	84.9
FEV_1_, % predicted	68.5	80.5	90.4	48.2	91.7
TLC, % predicted	58.7	61.3	72.4	78.4	80.9
6MWD, m	378	362	310	366	383
Initial SpO_2_, %	97	98	92	94	92
Lowest SpO_2_, %	93	93	84	82	86

RVSP= Right ventricular systolic pressure, sPAP= Systolic pulmonary arterial pressure, mPAP=mean pulmonary arterial pressure, PCWP= Pulmonary capillary wedge pressure, PVR= Pulmonary vascular resistance, CO= Cardiac output, 6MWD= 6- minuter walk distance

In total, four patients died during the study period. Three of these patients were females (all at stage IV) with evidence of PH; their deaths were attributed to respiratory failure. The fourth patient (stage II) was a male who was not subjected to echocardiography. The principal cause of death in this patient was pneumonia and respiratory failure.

## Discussion

To the best of our knowledge, the present work represents the first study to describe the clinical, physiological and radiological characteristics of PH in Arab patients with pulmonary sarcoidosis.

Pulmonary hypertension can complicate the course of patients with pulmonary sarcoidosis, and the presence of PH was recently identified as an important (negative) factor affecting survival.[5,6,20] The frequency of PH in our patients was 20.8%. The reported range of PH frequency in sarcoidosis patients is 5.7–73.8%, depending on the definition of PH, diagnostic methodology, patient ethnicity, institution and world region.[6,20–24] Interestingly, the gender distribution among sarcoidosis patients with PH varies among world regions. Patients from Japan[23] and Europe[20] are more likely to be men, whereas Americans with PH[21,22,24] are generally women. In the current study of Arab sarcoidosis patients, 85% of those found to have PH were women. In the present study, patients with PH were significantly more symptomatic than were those without PH. This finding stands in contrast to the results of Sulica and colleagues,[24] who found no significant difference in presenting complaints between patients with and without PH. Moreover, our data demonstrate that physiologic parameters, including FVC, FEV_1_ and TLC were significantly lower among patients with PH, consistent with previous studies.[21,23,24] Notably, 15% of patients with PH displayed near-normal pulmonary function test data, in keeping with previous reports of a lack of association between static measures of lung function and PH.[22,25] Several factors, including respiratory status, cardiac involvement, skeletal muscle weakness, fatigue and depression lead to exercise intolerance, which manifests as a reduced walking distance among sarcoid patients.[7,21,26–28] In the present study, a shorter walking distance in the PH group, with a trend toward statistical significance, was noted compared to patients without PH. Moreover, in the PH group oxygen saturation at rest and after exercise were markedly lower than in patients without PH, in agreement with the earlier study of Bourbonnais and Samavati.[21] Interestingly, 9 of 35 patients (25.7%) had normal echocardiography results despite showing significant oxygen desaturation on the 6MWT. Within this group, only two of nine patients (22.2%) were of advanced radiographic stage. Furthermore, these patients demonstrated significant impairment in physiologic parameters compared to patients without oxygen desaturation (data not shown). In addition, we compared those patients with the PH group and found no significant difference in clinical, physiological or radiographic findings (data not shown). This may be because echocardiography underestimates the presence of PH. Bourbonnais and colleagues reported that 32% of their patients with PH would have been overlooked had the diagnosis been made solely on the basis of echocardiographic data. All seven patients who displayed desaturation to <90% during the 6MWT were shown to have PH using RHC.[21] Another potential explanation is that such patients may represent a subset of patients with underlying exercise-induced PH leading to oxygen desaturation during the 6MWT. However, the value of routine exercise testing during RHC is unknown.

Comparison of chest radiographic findings in the present study showed that nearly half of our PH patients had radiographic evidence of fibrosis, consistent with previous studies.[20,21,23,24] and 55% developed PH in the absence of fibrotic changes, as also reported earlier.[20,23,24] These data, together with the near-normal physiologic parameters observed in 15% of our patients with PH, support the theory that mechanisms other than fibrosis are responsible for the development of PH.

The mechanism underlying PH development in sarcoidosis remains to be established. Processes suggested to date include granulomatous involvement of pulmonary veins manifesting clinically as pulmonary veno-occlusive disease, extrinsic compression by mediastinal or hilar adenopathy, cardiac involvement including systolic or diastolic dysfunction, increased production of vasoactive endothelin-1 and downstream effects of hypoxemia.[20,25,29–31] Because of the plethora of possible mechanisms and a lack of understanding of the precise pathogenesis, PH related to sarcoidosis falls into group 5 (the ‘miscellaneous’ category) in the recent clinical classification of PH.[32]

Possible benefits of corticosteroids for patients with sarcoidosis and PH are currently controversial.[20,22,25,33] Although all patients with PH were maintained on long-term corticosteroid therapy in the present study, it appears that this form of therapy is of limited value in the context of sarcoidosis and PH. Further prospective studies are warranted to explore the effects of corticosteroids and other anti-inflammatory therapy in patients with PH complicating sarcoidosis, separated into groups with respect to the presence of fibrotic or nonfibrotic lung disease.

Conventional CT and HRCT have provided essential information for evaluating various interstitial lung diseases, including sarcoidosis. However, according to the ATS/ERS/WASOG statement on sarcoidosis, CT scanning is indicated in patients yielding either normal or atypical chest radiography findings, or when specific complications are suspected.[11] Only a limited number of studies have examined CT appearance in the context of sarcoidosis and PH.[20,23] Recently, Nunes and associates reported extrinsic compression of the pulmonary artery caused by lymph node enlargement in 21.4% of patients with lung fibrosis secondary to sarcoidosis.[20] In the present study, we found no significant difference in mediastinal and/or hilar lymph node enlargement when we compared patients with and without PH, consistent with an earlier report by Handa and colleagues.[23] Not surprisingly, a majority (76.9%) of our patients with PH had CT patterns indicating fibrosis, whereas only 44.2% of patients without PH had such CT patterns. Furthermore, and extending the findings of Handa and colleagues,[23] the thickening of bronchovascular bundles (a potential mechanism for development of PH through extrinsic compression of the pulmonary artery) was not significant in the present study among patients with PH compared to those without PH. CT-pathologic correlations of ground-glass opacity have been variably associated with granulomatous infiltration or fibrosis.[34,35] Furthermore, conflicting statements on outcomes of patients with ground-glass attenuation are apparent when studies are compared.[36–38] In the present work, we found that the frequency of ground-glass opacity was significantly higher among patients with PH compared to those without PH, in agreement with the data of Nunes and associates.[20] It is unclear whether ground-glass opacity signifies pulmonary veno-occlusive disease[20] or rather reflects the presence of fine fibrosis.[39] At present, the clinical significance of ground-glass opacity in the context of sarcoidosis and PH remains unknown. Further large-scale studies are needed to explore the reversibility of these patterns using various PH-targeted therapies.

The case fatality rate from sarcoidosis ranges from 1 to 5%.[11] The cause of death varies with geographic location, ethnicity and gender. Patients from Japan are more likely to die from cardiac involvement, whereas most patients from other parts of the world die from respiratory failure.[11] In the present study, all four deaths were attributed to respiratory failure. However, three patients with fibrosis had evidence of underlying PH. Although patients at advanced radiographic stages are at higher risk of developing pulmonary complications, unfavorable prognostic factors were seen in subgroups of patients whose disease course was complicated by PH.[5,6,22] Further studies are needed to address the outcomes of fibrotic and nonfibrotic pulmonary sarcoidosis complicated by PH, with respect to treatment using PH-targeted therapy.

The present study had several limitations. First, our work is a retrospective review wherein clinical information was obtained from hospital records. The interval between radiography (including CT scanning) and echocardiography varied among patients. Our clinical setting is a tertiary-care hospital where selection bias toward patients with more advanced disease is inevitable. Only a limited number of patients in the present study underwent RHC. As RHC is the gold standard for PH diagnosis, overestimation of PH in the presence of parenchymal lung disease[40] is possible. However, we believe that echocardiography underestimated the presence of PH in our cohort, in line with the observations of Bourbonnais and colleague.[21] Finally, certain parameters were not measured in some patients, which precluded the use of additional tests to identify variables predicting the presence of PH.

In conclusion, PH represents a complex pathophysiologic disease, and can complicate the course of patients with pulmonary sarcoidosis. The frequency of PH in the present study was 20.8%. Patients with PH were found to be more likely to be symptomatic, showing marked impairment in physiologic studies, compared to those without PH. CT findings including fibrosis and ground-glass opacity are frequently encountered in patients with sarcoidosis-associated PH. Moreover, the 6MWT and Doppler echocardiography appear to be useful screening tools in the context of sarcoidosis with PH. Our study supports the notion that PH can occur at various radiographic stages, and careful attention to development of this complication is essential, as PH appears to be associated with poor clinical outcomes.
